# The GLP-1 Receptor Agonist Exendin-4 and Diazepam Differentially Regulate GABA_A_ Receptor-Mediated Tonic Currents in Rat Hippocampal CA3 Pyramidal Neurons

**DOI:** 10.1371/journal.pone.0124765

**Published:** 2015-04-30

**Authors:** Sergiy V. Korol, Zhe Jin, Bryndis Birnir

**Affiliations:** Department of Neuroscience, Uppsala University, Uppsala, Sweden; Dalhousie University, CANADA

## Abstract

Glucagon-like peptide-1 (GLP-1) is a metabolic hormone that is secreted in a glucose-dependent manner and enhances insulin secretion. GLP-1 receptors are also found in the brain where their signalling affects neuronal activity. We have previously shown that the GLP-1 receptor agonists, GLP-1 and exendin-4 enhanced GABA-activated synaptic and tonic currents in rat hippocampal CA3 pyramidal neurons. The hippocampus is the centre for memory and learning and is important for cognition. Here we examined if exendin-4 similarly enhanced the GABA-activated currents in the presence of the benzodiazepine diazepam. In whole-cell recordings in rat brain slices, diazepam (1 μM), an allosteric positive modulator of GABA_A_ receptors, alone enhanced the spontaneous inhibitory postsynaptic current (sIPSC) amplitude and frequency by a factor of 1.3 and 1.6, respectively, and doubled the tonic GABA_A_ current normally recorded in the CA3 pyramidal cells. Importantly, in the presence of exendin-4 (10 nM) plus diazepam (1 μM), only the tonic but not the sIPSC currents transiently increased as compared to currents recorded in the presence of diazepam alone. The results suggest that exendin-4 potentiates a subpopulation of extrasynaptic GABA_A_ receptors in the CA3 pyramidal neurons.

## Introduction

Over a number of years evidence has accumulated suggesting that diabetes mellitus increases the risk of impairment of cognitive functions [1–5]. A number of mechanisms may be involved including decreased signaling by metabolic hormones. It is well established that the brain expresses receptors for many metabolic hormones, including receptors for insulin and the incretins, e.g. glucagon-like peptide-1 (GLP-1) [6]. It is, therefore, somewhat surprising that apart from the hypothalamus [7], we know relatively little about the effects of metabolic hormones on neurons and function of neuronal circuits and, thereby, brain function. In the brain, the hippocampus is a part of the medial temporal lobe and is the center for memory formation and learning [6, 8]. Receptors for metabolic hormones are prominently expressed in the hippocampus, including the GLP-1 receptor [6, 9]. It can be activated not only by the endogenous GLP-1, but also by compounds that mimic its action such as exendin-4 that is also known as the type 2 diabetes medicine exenatide. We have shown previously that activation of the GLP-1 receptor system alters GABA signaling in hippocampal CA3 pyramidal neurons [10].

Diazepam (Valium) is a benzodiazepine that is used to treat a broad spectrum of conditions including insomnia, anxiety and seizures [11]. It is a positive allosteric modulator of the GABA_A_ receptors and binds to the receptor at the interface between α and γ2/3 subunits in the pentameric receptor [12]. It potentiates GABA_A_ receptor activity at the single channel level by increasing the open probability and the single-channel conductance resulting in an apparent increase in the GABA_A_ receptor affinity for GABA [13–16]. GABA_A_ receptors are located at synapses in the postsynaptic neurons but also outside of synapses where they are called extrasynaptic or nonsynaptic GABA_A_ receptors. The synaptic receptors generate the phasic inhibitory postsynaptic currents (IPSCs) whereas the extrasynaptic receptors generate the long lasting tonic currents. Both types of the GABA-activated currents generally reduce neuronal excitability in mammalian brains [17, 18].

In the present study we examined in rat hippocampal CA3 pyramidal neurons the effects of diazepam applied alone or together with exendin-4 on the GABA_A_ receptor-mediated synaptic and tonic currents. In the neurons, diazepam enhanced the sIPSCs and doubled the tonic currents revealing a significant contribution of GABA_A_ receptors containing the γ2/3 subunit not only to synaptic but, importantly, also to tonic currents generated in the cells. Only for the tonic current were the effects of the drugs additive suggesting that exendin-4 potentiates a specific subpopulation of extrasynaptic GABA_A_ receptors expressed in the neurons. The results are consistent with independent modes of modulation by diazepam and exendin-4 of the GABA-activated currents in rat hippocampal CA3 pyramidal neurons.

## Materials and Methods

### Ethics statement

All procedures were approved by Uppsala Animal Ethical Committee, permit number C129/14.

### Brain slice preparation procedure

Wistar rats aged 16–22 days were used for hippocampal slice preparation. Animals were handled and sacrificed accordingly to the local ethical guidelines and approved animal care protocols by the Uppsala Animal Ethical Committee, Uppsala, Sweden (permit: C129/14). Hippocampal slices were prepared as previously described [19]. Briefly, the animal was decapitated, the brain rapidly removed and immersed into an ice-cold artificial cerebrospinal fluid (ACSF) containing (in mM): 124 NaCl, 3 KCl, 2.5 CaCl_2_, 1.3 MgSO_4_, 26 NaHCO_3_, 2.5 Na_2_HPO_4_ and 10 glucose with pH 7.3–7.4 when bubbled with 95% O_2_ and 5% CO_2_. Sagittal hippocampal slices 400 μm thick were prepared with a vibratome (Leica VT1200S) in ice-cold ACSF gassed with 95% O_2_ and 5% CO_2_. Slices were incubated in ACSF at 37°C for 1 h and stored at room temperature (20–22°C).

### Recording and analysis of electrophysiological data

All patch-clamp recordings were done at room temperature (20–22°C). Most of the chemicals were purchased from Sigma-Aldrich (Germany) or Anaspec (exendin-4; USA). Bicuculline methiodide from Santa Cruz Biotechnology (USA) or Sigma-Aldrich (Germany) was used. The pipette solution contained (mM): 140 CsCl, 1 CaCl_2_, 3 EGTA, 0.5 KCl, 1 MgCl_2_, 2 ATP-Mg, 0.3 GTP-Na, 5 QX-314 bromide, 10 TES; pH 7.25 with CsOH. The recording pipettes were made from borosilicate glass capillaries (Harvard Apparatus; UK) with DMZ-Universal Puller (Zeitz Instruments; Germany) and had resistance of 2 to 4 MΩ when filled with the pipette solution. The holding potential (V_h_) was set to −60 mV and used in all experiments. ACSF containing kynurenic acid (3 mM) and other drugs was continuously perfused through the experimental chamber (perfusion rate of 2 mL/min) during the experiments. Electrophysiological recordings were done using Axopatch 200B amplifier (Molecular Devices; USA), filtered at 2 kHz, sampled at 10 kHz by analogue-to-digital converter, Digidata 1322A (Molecular Devices; USA), and stored in a computer. The recordings were analyzed with pClamp 10 (Molecular Devices; USA) and MiniAnalysis 6 (Synaptosoft, Inc.; USA) software. The amplitude of the tonic current was defined as the difference between the baseline current levels before and after the drug application [20]. The control for the drug effects on the frequency of the sIPSCs was the period of recording immediately before the first drug application that contained 190 to 220 events (normally, 20 to 90 s). sIPSC frequency at the maximal drug effect was calculated and normalized to its control value in the same cell. The average value of the baseline current during the transient change in the current value during exendin-4 application was fitted with a double exponential function (Eq 1): *y = y*_*0*_ *+ A*_1_*exp(–t/τ_*rise*_ *) − A*_*2*_ *exp(–t/τ_*decay*_ *)*, where *y*_*0*_, *A*_*1*,*2*_ are arbitrary constants; and the *τ*_*rise/decay*_ are time constants for the rise and the decay phase of the transient current, respectively.

### Statistical analysis

Statistical analysis was carried out using SigmaPlot 11 (Systat Software; USA), MiniAnalysis 6 (Synaptosoft, Inc.; USA) or GraphPad Prism 6 (GraphPad Software; USA) software. Results are presented as mean ± standard error of the mean (SEM). Paired Student's t-test was used for data-sets treated as pairs and normally distributed. The Tukey method was used to detect outliers. Statistical analysis was performed after excluding outliers. Non-parametric Mann-Whitney test was used for data-sets that were not normally distributed. The significance level was set at P < 0.05.

## Results

### Effects of diazepam and exendin-4 on synaptic and tonic GABA_A_ receptor-mediated currents in hippocampal CA3 pyramidal neurons

**Fig 1:**
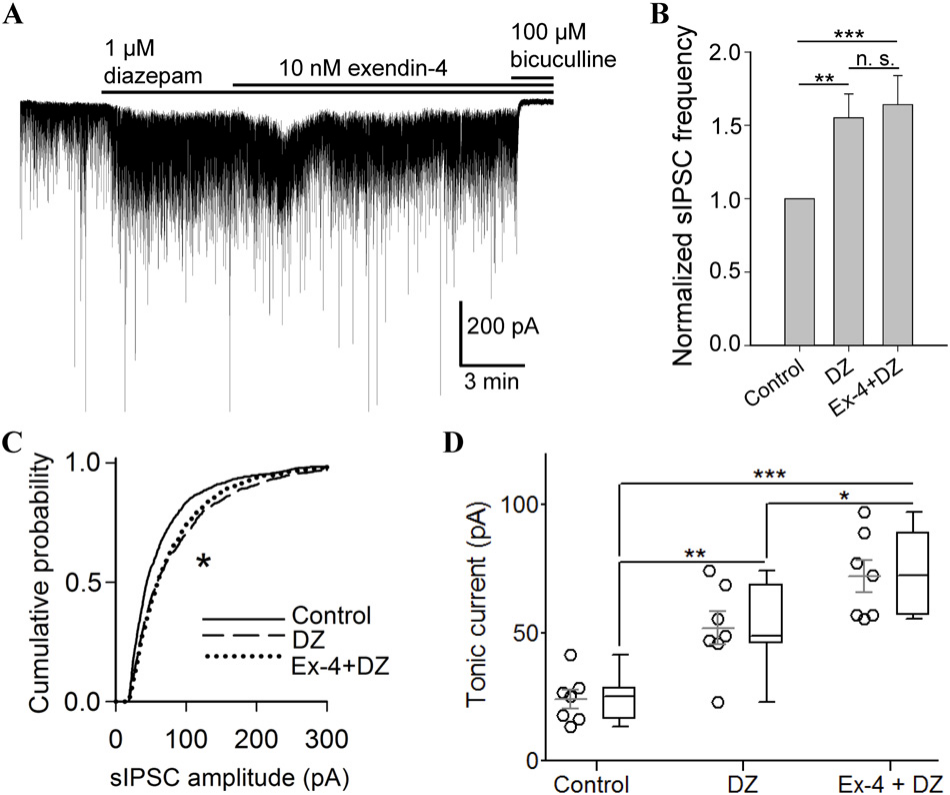
Diazepam alone and co-applied together with exendin-4 potentiate spontaneous inhibitory postsynaptic currents (sIPSCs) and the tonic GABA_A_ receptor-mediated current. (**A**) Diazepam (1 μM) induces sustained increase in tonic and sIPSCs, and its co-application with exendin-4 (10 nM) leads to additional but transient enhancement in tonic current. Horizontal black bars show the time of application of the drugs. (**B**) sIPSC frequencies increased upon diazepam application alone and together with exendin-4. **P < 0.01, ***P < 0.001, n = 7 (non-parametric Mann-Whitney test). n. s., not significant. (**C**) Cumulative probability histograms of sIPSC amplitudes for diazepam and its co-application with exendin-4 revealed increase of sIPSC amplitudes at both conditions comparatively to control. *P < 0.05, n = 7 for both conditions. No difference was detected between DZ and Ex-4+DZ. (**D**) Tonic currents in individual neurons at diazepam administration and its co-perfusion with exendin-4. Data from each group is presented as a scatter dot plot with mean ± SEM and a box and whiskers plot with median values plotted by Tukey method. No outliers were detected. *P < 0.05, **P < 0.01, ***P < 0.001, n = 7 (Student’s t-test). DZ, diazepam; Ex-4+DZ, co-application of exendin-4 and diazepam.

We examined the effects of diazepam (1 μM) and exendin-4 (10 nM) on sIPSCs and tonic GABA_A_ receptor-mediated currents recorded in the rat hippocampal CA3 pyramidal neurons. Bicuculline (100 μM), a GABA_A_ receptor antagonist, was added at the end of each recording to inhibit the GABA-evoked synaptic and tonic currents. Fig 1 shows the effects of first adding diazepam and then, diazepam plus exendin-4. Diazepam alone increased the frequency of the synaptic currents by a factor of 1.6 as compared to control (Fig 1A and 1B). The most frequent peak amplitude of the sIPSCs was also enhanced by diazepam and increased by a factor of 1.3 (Fig 1A and 1C) as well as the tonic current which increased from 24 ± 4 pA (n = 7) in control to 52 ± 6 pA (n = 7; Fig 1A and 1D). The enhanced level of the tonic current was maintained as long as diazepam was applied (Fig 1A).

We then examined the effect of exendin-4 on the sIPSCs and the tonic current in the presence of diazepam. We have previously shown that 10 nM exendin-4 enhanced the frequency but not the amplitude of the sIPSCs and transiently potentiated the tonic currents in the CA3 pyramidal neurons [10]. After the initial perfusion with diazepam, exendin-4 plus diazepam was applied to the brain slices. Exendin-4 in the presence of diazepam did not increase the frequency of the sIPSCs above what was observed when diazepam alone was applied (Fig 1A and 1B) and similarly, no further increase was recorded in the sIPSC amplitude when exendin-4 was applied with diazepam (Fig 1A and 1C). In contrast, on exposure to exendin-4, an additional but transient increase in the tonic current was recorded and the peak value was on the average 72 ± 6 pA (n = 7; Fig 1A and 1D). Interestingly, the additional increase of the tonic current when exendin-4 was applied together with diazepam, is similar in magnitude (~23 pA) as the recorded peak tonic current in exendin-4 alone [10].

### The time course of the transient tonic current evoked by exendin-4 is modulated by diazepam

**Fig 2:**
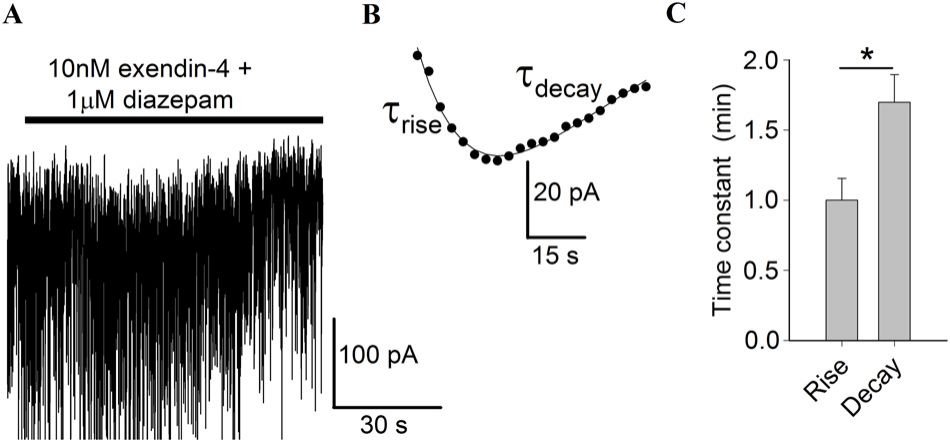
The kinetics of the transient tonic current induced by application of exendin-4 in the presence of diazepam. (**A**) A representative example of the transient current evoked by co-application of exendin-4 (10 nM) and diazepam (1 μM). Horizontal black bar indicates time of co-application of the two drugs. Before that time hippocampal slice was bathed in diazepam alone. (**B**) A fit to the transient current by Eq 1: *y = y*_*0*_ *+ A*_1_*exp(–t/τ_*rise*_ *) − A*_*2*_ *exp(–t/τ_*decay*_ *)*. (**C**) Values of time constants *τ*_*rise*_ and *τ*_*decay*_ at simultaneous application of two drugs. *P < 0.05, n = 6 for both time constants (Student’s t-test).

During the first minutes of the exendin-4 administration in the continuous presence of diazepam the tonic current transiently increased (Figs 1A and 2A). The rising and decay phase of the current could be fitted with a bi-exponential function to examine whether the combination of drugs affects the activation or the decay phase of the current. We fitted the time course of the transient increase of the baseline current with Eq 1 (Fig 2B). In the presence of both drugs, exendin-4 and diazepam, the baseline current increased with a characteristic time constant *τ*_*rise*_ = 1.0 ± 0.2 min (n = 6) which was significantly smaller than the decay time constant, *τ*_*decay*_ = 1.7 ± 0.2 min (n = 6, Student's t-test: P = 0.019) (Fig 2C). We have previously reported that both the rising and the decay phase of the transient tonic current evoked with exendin-4 alone in the CA3 pyramidal neurons can be fitted with time constants of about 2 min [10]. In this study in the presence of 1 μM diazepam, the current rate of rise was faster (i.e. time constant decreased) but the decay was similar to currents evoked in exendin-4 alone.

## Discussion

In recent years metabolic hormones have emerged as important modulators of hippocampal function [10, 21–23]. Here exendin-4, a mimetic of the metabolic hormone glucagon-like peptide-1 (GLP-1), and the benzodiazepine diazepam both potentiated GABA_A_ receptor-mediated currents in rat hippocampal CA3 pyramidal neurons. These cells constitute an important part of the hippocampal neuronal network that is important for memory formation [8]. The CA3 neuronal activity is normally regulated by GABA-releasing inhibitory hippocampal interneurons. Our results show that in the presence of diazepam, exendin-4 does not enhance the sIPSCs above what was observed in diazepam alone. In contrast, the tonic current was enhanced by both drugs and the effects were additive. Interestingly, diazepam enhances receptors containing the γ2 or γ3 subunits normally localized at synapses but clearly also having significant extrasynaptic distribution in the CA3 pyramidal neurons. The results obtained with exendin-4 are furthermore consistent with that exendin-4 enhances a subpopulation of extrasynaptic GABA_A_ receptors.

The drugs enhance the GABA signaling by different mechanisms. Diazepam interacts directly with the receptor by binding to a binding site on the GABA_A_ receptors [24–26]. Upon application of diazepam alone, the tonic current doubled in amplitude and the frequency and the amplitude of the sIPSCs increased in comparison to control sIPSCs values. It is well established that diazepam can enhance synaptic currents [27], and GABA_A_ receptors containing the γ2 or 3 subunit can form extrasynaptic receptors [11, 28] and thus be potentiated by diazepam. Since exposure to diazepam resulted in the tonic current doubled in size as compared to control, it suggests that a large proportion of the extrasynaptic GABA_A_ receptors in the CA3 pyramidal neurons contain the γ2 or perhaps the γ3 subunit.

Exendin-4, on the other hand, activates GLP-1 receptors in pre- and postsynaptic neurons [10]. Activation of GLP-1 receptors then results in increased release of GABA from presynaptic terminals and activation of intracellular signaling in postsynaptic cells resulting in enhanced GABA_A_ receptor function that is manifested in transiently increased sIPSC frequency and amplitude of the tonic current [10]. In the present work, tonic but not synaptic currents were enhanced when exendin-4 was co-applied with diazepam after a prolonged perfusion of the slices with diazepam alone. At the synapse, diazepam increases the apparent affinity of the GABA_A_ receptors for GABA whereas exendin-4 increases the concentration of GABA in the synaptic cleft [10, 11, 14]. Hence, only as long as the GABA_A_ receptors are not saturated with GABA will co-application of diazepam and exendin-4 be expected to increase the sIPSC amplitude [10, 27].

The CA3 pyramidal neurons normally exhibit significant tonic current in contrast to the hippocampal CA1 pyramidal neurons [10, 29, 30]. The tonic current in the presence of diazepam in this study was about twice the size of the current without drugs and, interestingly, transiently increased when exendin-4 was added. The increase by exendin-4 is similar in amplitude to the tonic current evoked by exendin-4 alone. The additive effects of diazepam and exendin-4 on the amplitude of the tonic current and then the return to the diazepam-enhanced current level, at the end of the transient current increase by exendin-4, supports the notion that exending-4 enhances a subset of extrasynaptic GABA_A_ receptors in the CA3 pyramidal neurons.

GABA_A_ receptors containing the γ2 or 3 subunit are potentiated by benzodiazepines [31] and are prominently expressed at synapses. That γ2/3 GABA_A_ receptor subtypes also contribute to tonic currents has been reported see e.g. [28, 32, 33] in addition to the current study on rat CA3 pyramidal neurons. This is in contrast to rat hippocampal dentate gyrus neurons where the δ subunit is a prominent component of the extrasynaptic receptors [28, 34]. The principal cells that make up the basic neuronal network in the hippocampus are the dentate gyrus granule cells, the CA3 and CA1 pyramidal neurons. These cell-types differ in the basal level of the tonic current and the subtypes of GABA_A_ receptors expressed in the cells. Whether they also differ in the level or type of modulation of the GABA signaling by metabolic hormones is still being explored [6, 29, 35,10]. Since these neurons form a crucial part of the hippocampal neuronal network and the hippocampus is the centre for memory and learning, metabolic hormones can be expected to have impact on hippocampal function like memory formation and cognition.
